# A novel method for prokaryotic promoter prediction based on DNA stability

**DOI:** 10.1186/1471-2105-6-1

**Published:** 2005-01-05

**Authors:** Aditi Kanhere, Manju Bansal

**Affiliations:** 1 Molecular Biophysics Unit, Indian Institute of Science, Bangalore 560 012, India

## Abstract

**Background:**

In the post-genomic era, correct gene prediction has become one of the biggest challenges in genome annotation. Improved promoter prediction methods can be one step towards developing more reliable *ab initio *gene prediction methods. This work presents a novel prokaryotic promoter prediction method based on DNA stability.

**Results:**

The promoter region is less stable and hence more prone to melting as compared to other genomic regions. Our analysis shows that a method of promoter prediction based on the differences in the stability of DNA sequences in the promoter and non-promoter region works much better compared to existing prokaryotic promoter prediction programs, which are based on sequence motif searches. At present the method works optimally for genomes such as that of *Escherichia coli*, which have near 50 % G+C composition and also performs satisfactorily in case of other prokaryotic promoters.

**Conclusions:**

Our analysis clearly shows that the change in stability of DNA seems to provide a much better clue than usual sequence motifs, such as Pribnow box and -35 sequence, for differentiating promoter region from non-promoter regions. To a certain extent, it is more general and is likely to be applicable across organisms. Hence incorporation of such features in addition to the signature motifs can greatly improve the presently available promoter prediction programs.

## Background

Accumulation of a huge amount of genome sequence data in recent years and the task of extracting useful information from it, has given rise to many new challenges. One of the biggest challenges is the task of gene prediction and to fulfil this need, several gene prediction programs have been developed (For reviews see [1-5]). Most of these prediction programs require training based on prior knowledge of sequence features such as codon bias, which in turn are organism specific. In such cases, lack of large enough samples of known genes, as typically seen in a newly sequenced genome, can lead to sub optimal predictions. On the other hand, some gene prediction methods are based on the homology between two or more genomes but these methods are not of much help for gene prediction in case of genomes with no homologues. In addition, most of the gene prediction programs concentrate on the protein-coding regions and RNA genes, that can make up to 5 % of total protein coding genes, are neglected. Hence it is important to design *ab initio *gene prediction programs. One of the important steps towards *ab initio *gene prediction is to develop better promoter and TSS (transcription start site) prediction methods.

Although reasonable progress has been achieved in the prediction of coding region, the promoter prediction methods are still far from being accurate [6-9] and there are some very obvious reasons for these inaccuracies. One of the major difficulties is that the regulatory sequence elements in promoters are short and not fully conserved in the sequence; hence there is a high probability of finding similar sequence elements elsewhere in genomes, outside the promoter regions. This is the reason why most of the promoter prediction algorithms, which are based on finding these regulatory sequence elements, end up predicting a lot of false positives. Thus it is likely that incorporation of additional characteristics, which are unique to the promoter region, will help in improving the currently available promoter prediction methods.

In our earlier analysis, we observed that in case of bacteria as well as in eukaryotes, various properties of the region immediately upstream of TSS differ from that of downstream region [10]. There are differences in sequence composition as well as in different sequence dependent properties such as stability, bendability and curvature. The upstream region is less stable, more rigid and more curved than downstream region. Some of these observations are supported by other studies carried out independently on genomic sequences [9,11-17]. Among all types of promoters, the most prominent feature is the difference in DNA duplex stabilities of the upstream and downstream regions. Here, we propose a prokaryotic promoter prediction method, which is based on the stability differences between promoter and non-promoter regions.

## Results and discussion

### Lower stability of promoter regions in bacterial sequences

**Figure 1:**
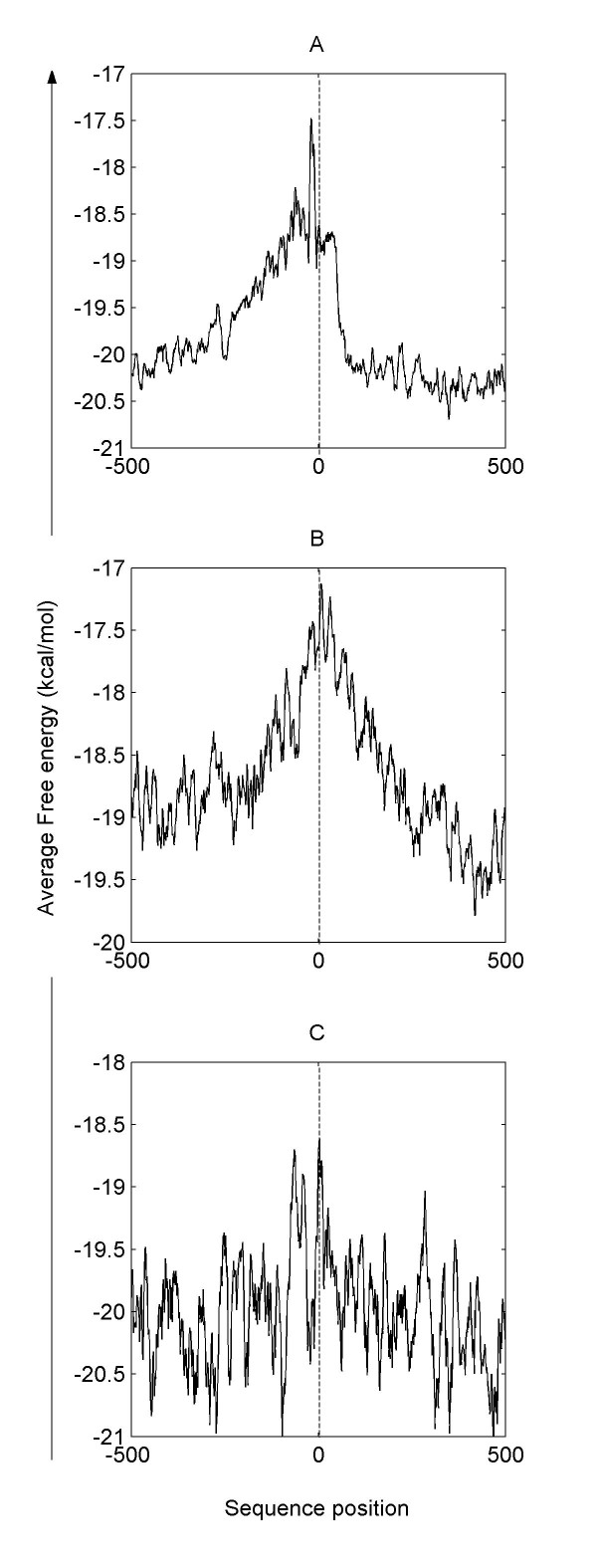
**Overall free energy profile around bacterial TSS **The figure shows the average free energy profiles of A) *Escherichia coli *(227 promoters) and B) *Bacillus subtilis *(89 promoters) C) *Corynebacterium glutamicum *promoters (28 promoters). The profiles extend from 500 nt upstream to 500 nt downstream of transcription start site (positioned at 0, shown as dashed line). The nucleotide sequence position is shown on x-axis. More negative values of free energy indicate greater stability.

It is well known that the stability of a DNA fragment is a sequence dependent property and depends primarily on the sum of the interactions between the constituent dinucleotides. The overall stability for an oligonucleotide can thus be predicted from its sequence, if one knows the relative contribution of each nearest neighbour interaction in the DNA [18]. The average stability profiles for three sets of bacterial promoter sequences calculated (using 15 nt moving window) based on this principle is shown in Figure 1. It is interesting that the promoters from diverse bacteria, which have quite different genome composition (A+T composition: *E. coli *0.49, *B. subtilis *0.56 and *C. glutamicum *0.46), show strikingly similar features. Promoters from all the three bacteria show low stability peak around the -10 region. The second prominent feature in the free energy profiles of all the three bacteria is the difference in stabilities of the upstream and downstream regions. In all the three groups of promoter sequences, the average stability of upstream region is lower than the average stability of downstream region. But the three sets of promoter sequences differ in their basal energy level, which seems to be dependent on the nucleotide composition of the bacteria.

### Detailed analysis of *E. coli *promoter sequences

**Figure 2:**
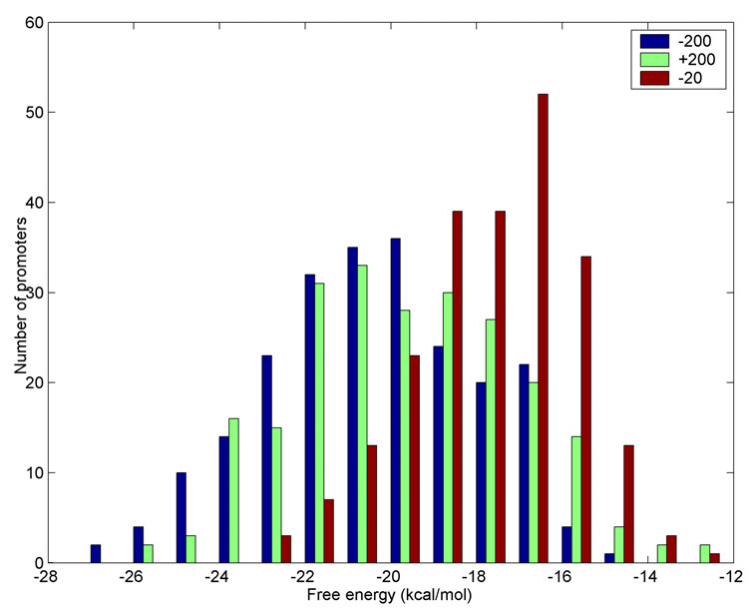
**Histogram showing the free energy distribution corresponding to upstream region (-200), promoter region (-20) and coding region (+200) in *E. coli *sequences **The free energy distribution corresponding to position -20 (calculated for a 15 nt window extending from -20 to -6) is shown as brown bars. Free energy distribution corresponding to positions -200 (calculated for a 15 nt window from -200 to -186, shown in green bars) and +200 (calculated for 15 nt window from +200 to +214, shown in blue bars) are also shown for comparison. Each bar corresponds to 1 kcal/mol. The average free energies corresponding to -20, -200 and +200 positions are -17.48 kcal/mol, -19.42 kcal/mol and -20.19 kcal/mol respectively.

In order to get a better insight into the stability feature, we carried out a detailed analysis of *E. coli *promoter sequences. Our statistical analysis using "Wilcoxon signed test for equality of medians" (see METHODS) shows that the free energy distribution corresponding to a fragment extending from position -148 to 51 in the *E. coli *sequences is appreciably different from the energy distribution calculated in randomly selected windows, at a significance level as high as 0.0001. A comparison of free energy distribution at position -20 (corresponding to the promoter region) with distributions at positions -200 (corresponding to the region upstream of promoter region) and +200 (corresponding to the coding region) is shown in Figure 2. It is clearly seen that the region immediately upstream of TSS is much less stable than the other two regions. The average free energy at -20 position is -17.48 kcal/mol while average free energies at the -200 and +200 positions are -19.42 kcal and -20.19 kcal/mol respectively. The Kolmogorov-Smirnov test also confirms that the free energy distribution at position -20 significantly differs from that at -200 and +200 positions at a very high significance level (alpha = 10^-10^).

### Details of methodology

This difference in free energy and the stability of promoter regions as compared to that of coding and other non-coding regions can be used to search for the promoters. Based on this consideration, a new scoring function D(n) is defined, which will look for differences in free energy of the neighbouring regions of position n:

D(n) = E1(n) - E2(n)

where,


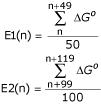


**Figure 3:**
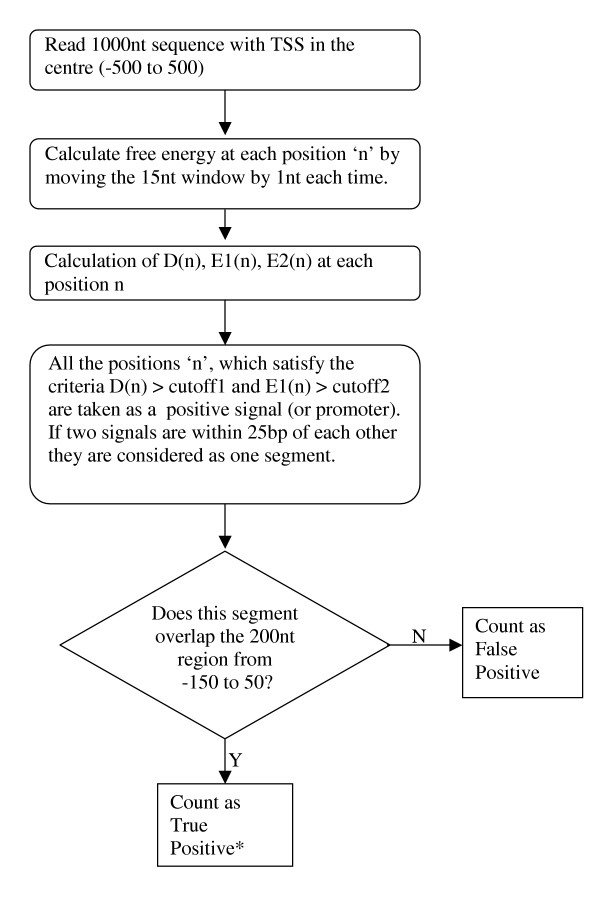
A flowchart summarizing our methodology * If there are more than one predictions in the 200 nt region (-150 to 50) then only one prediction which is nearest to the TSS is taken as a true prediction. The remaining predictions are counted as false predictions.

**Figure 4:**
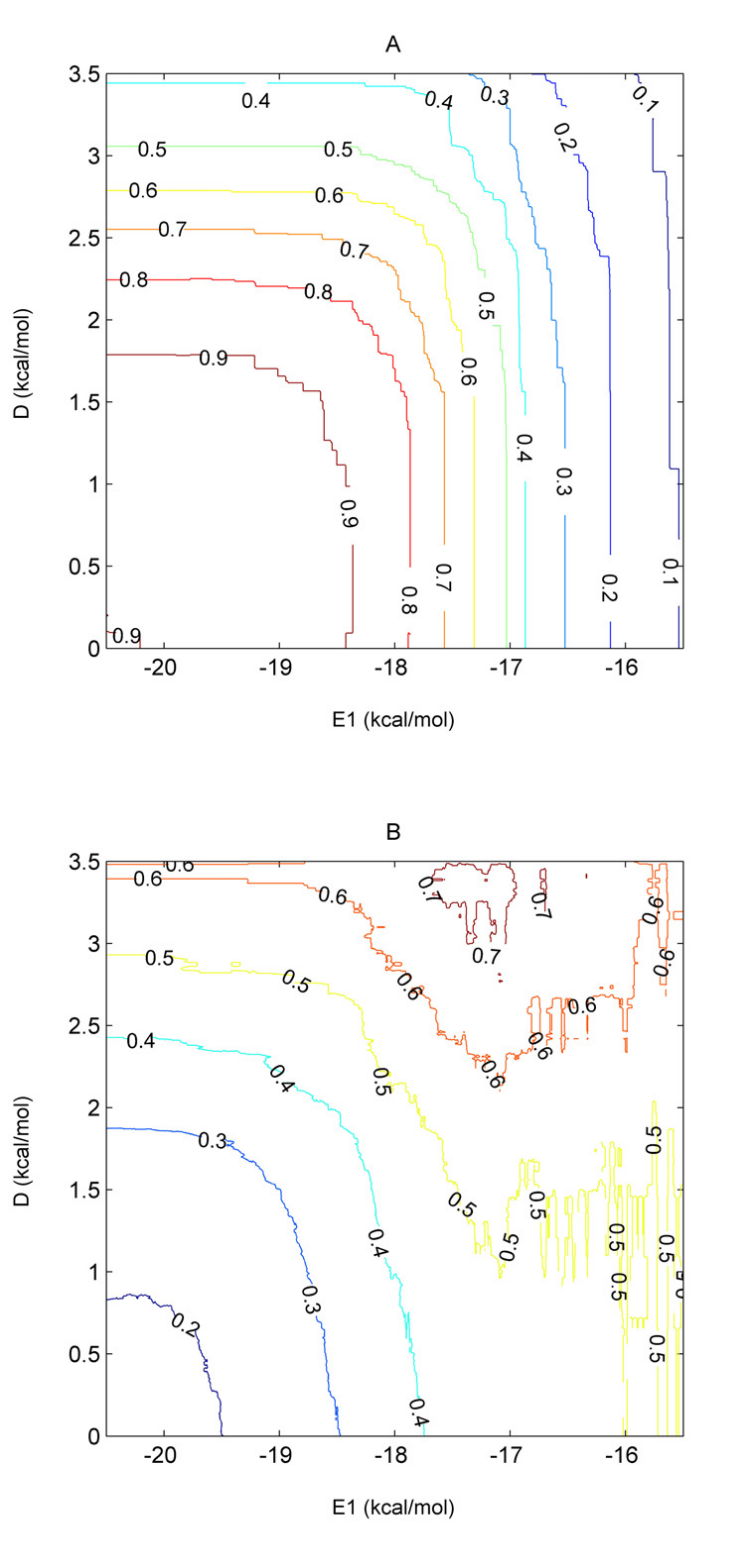
**Sensitivity and precision contour plots **The E1 value cut-offs are plotted on x-axis while D value cut-offs are plotted on y-axis. The different A) sensitivity and B) precision levels are shown by colours ranging from dark blue to brown, where dark blue corresponds to lowest value and brown corresponds to highest value.

**Figure 5:**
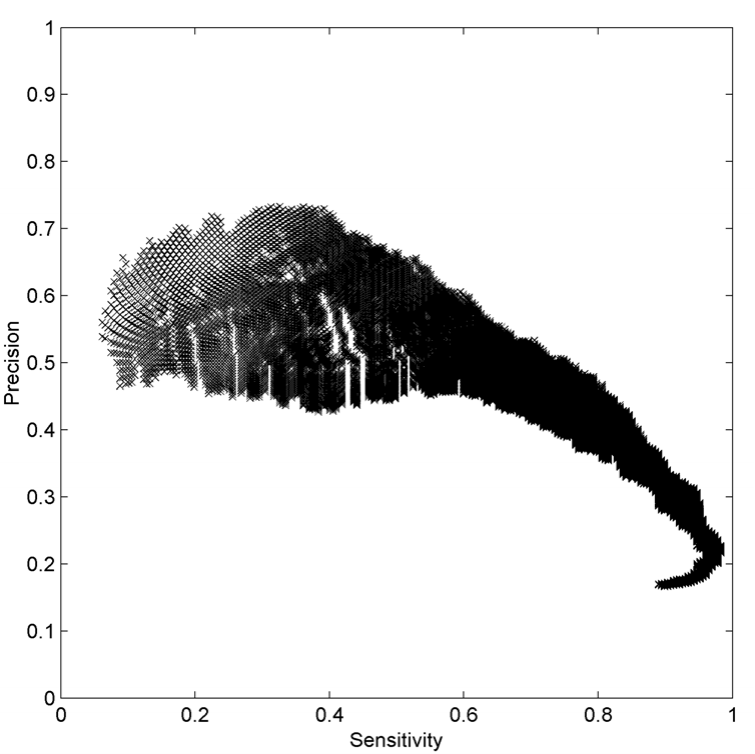
**A plot showing range of precision values obtained for a given sensitivity **The sensitivity (x-axis) and precision (y-axis) corresponding to different E1 and D cut-offs has been plotted.

Thus, E1(n) and E2(n) represent the free energy (see METHODS) average in the 50 nt region starting from nucleotide n and neighbouring 100 nt region starting from nucleotide n+99, respectively. The E1 value represents the basal energy level, which is characteristic of the given bacterial genome (e.g. in this case *E. coli*) and the D value represents the free energy difference in the two neighbouring regions. A stretch of DNA is assigned as promoter only if the average free energy of that 50 nt region (E1) and difference in free energy as compared to its neighbouring region (D) is greater than the chosen cut-offs. The protocol followed to calculate the true and false positives and hence sensitivity and precision is presented in the form of a flowchart in Figure 3. Identical sensitivity values can be achieved using different combinations of D and E1 cut-off values, which is obvious from the contour plot shown in Figure 4A. Similarly, different combinations of D and E1 cut-offs can lead to similar precisions (Figure 4B). But we observe that the use of different D and E1 cut-offs, corresponding to a given sensitivity level, results in a wide range of precisions (Figure 5). Hence, in order to attain a desired level of sensitivity the D and E1 cut-off values are chosen such that the number of false positives is minimum and the precision is maximum.

**Figure 6:**
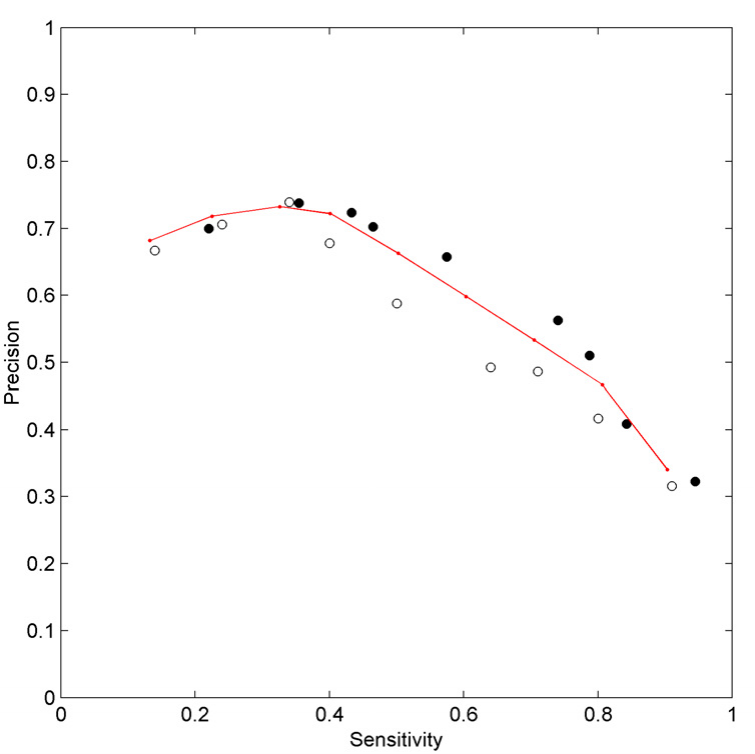
**The comparison of sensitivity and precision values from test and 'training' sets **The sensitivity (x-axis) and precision (y-axis) corresponding to 1) test set (filled circles), 2) training set (open circles) and 3) the whole *E. coli *dataset (red) is shown. The sensitivity and precision values for the test set were calculated using E1 and D cut-offs derived from the training set.

Initially, we divided the *E. coli *sequence data into two sets. The E1 and D cut-off values corresponding to different sensitivity levels were obtained for 100 randomly selected sequences (1^st ^set). These cut-off values were then applied to a second set consisting of remaining 127 sequences. The sensitivity and precision values calculated for the first and second set match very well. We also found that very similar results can be obtained when we use the whole dataset (Figure 6). Hence, we present the results for the whole dataset rather than separately for two sets. The D and E1 cut-offs and the number of false positives corresponding to different levels of sensitivity are given in Table 1. To confirm the validity of our choice, we used another set of 1000 nt long sequences extracted from the centre of the ORFs, which were more than 2000 nt long. The results corresponding to this set of control fragments are also given in Table 1 and show very few false positives.

**Figure 7:**
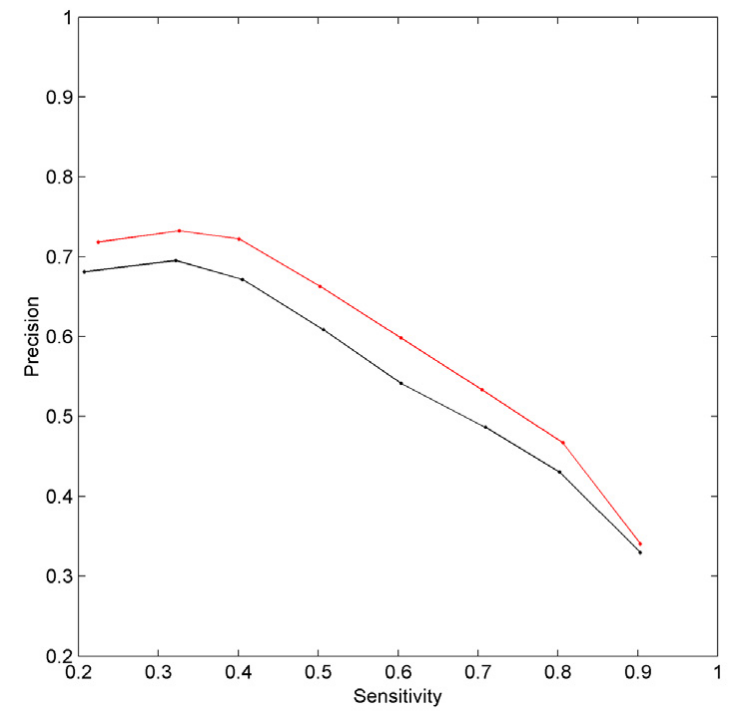
**Change in precision with the use of different sized windows for E2 calculation **The sensitivity (x-axis) and precision (y-axis) values corresponding to the use of 1) 50 nt window (black) and 2) 100 nt window (red) for E2 calculation.

In principle, D can also be calculated using equal sized windows, i.e. 50 nt, for both E1 and E2 instead of a 50 nt window for E1 and a 100 nt window for E2. However, our calculations show that use of equal sized windows, for E1 as well as E2 calculations, results in a slightly lesser precision than when 100 nt window is used for E2 calculations (Figure 7). Hence, in our promoter predictions, we chose a 100 nt window for E2 calculations.

### Comparison with other promoter prediction programs

**Figure 8:**
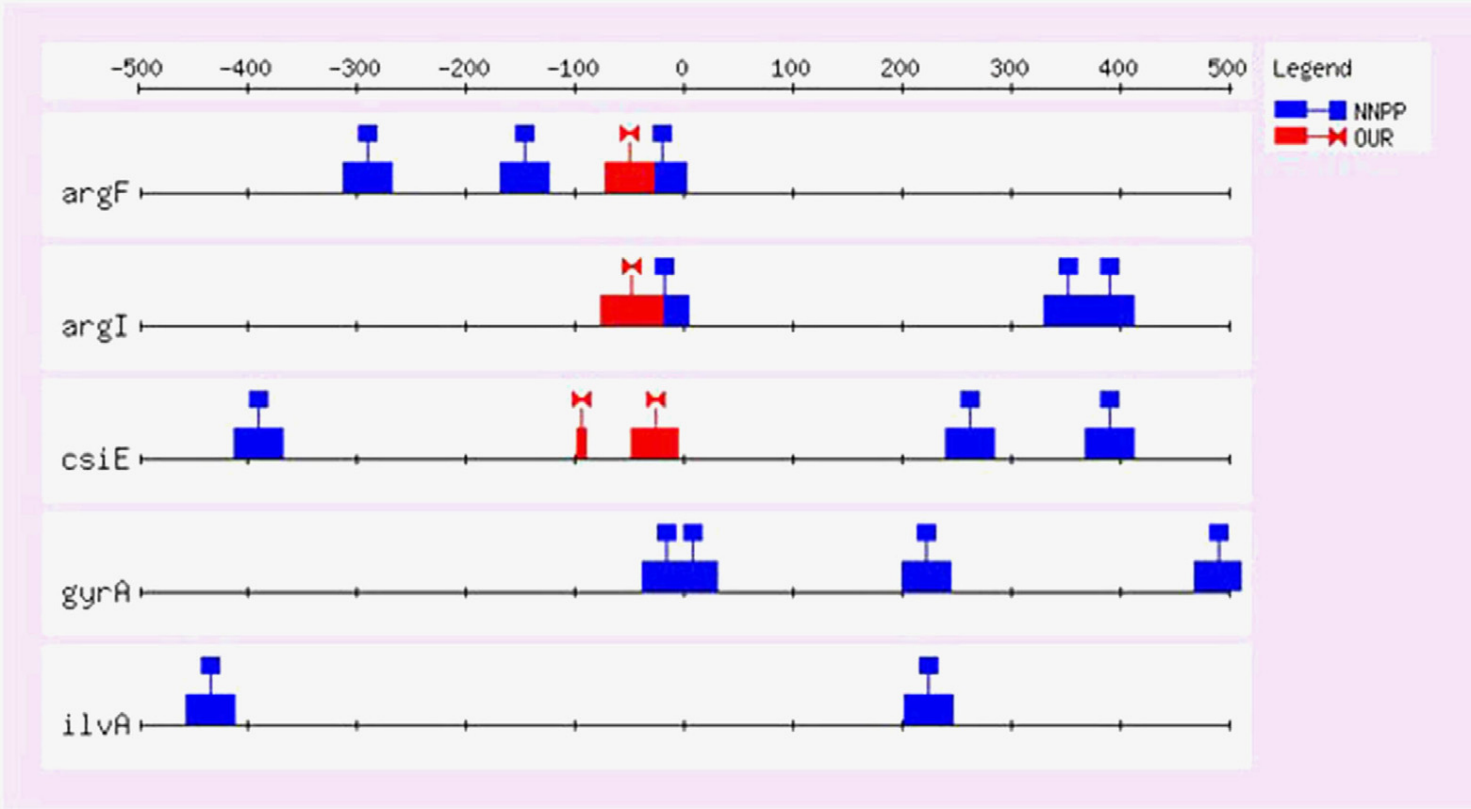
**Examples illustrating the predictions with our method as well as NNPP **The promoter predictions for the argF, argI, csiE, gyrA, ilvA genes by our method (red) as well as by NNPP (blue) in the 1000 nt fragments (-500 to 500) with the TSS at the centre. The figure is generated using FEATURE MAP program [39].

A large number of promoter prediction programs have been developed for eukaryotic sequences and are easily accessible, while NNPP [19,20] is the only available prokaryotic promoter prediction program. It is a neural network based method where prediction for each sequence element constituting promoter sequence is combined in time-delay neural networks for a complete promoter site prediction. Some other prokaryotic promoter prediction methods are based on weight matrix pattern searches [21-24]. One of the representative weight matrix method, proposed by Staden [21], uses three weight matrices corresponding to the -35 sequence, the -10 sequence and the transcription start site. It also takes into account the spacing between the -35 and -10 motifs, as well as the distance between the -10 motif and the transcription start site. A brief comparison of the results obtained by our method and the other two methods (Staden method and NNPP program) is given in Table 2. It can be clearly seen from Table 2 that for similar sensitivity, our program gives much better accuracy than the other two programs. It is pertinent to mention here that our method differs from the other two methods in one major respect, namely our method tries to find a promoter region while the other two programs try to pinpoint the transcription start site. It may be argued that the lesser number of false positives in our prediction method, as compared to the other two algorithms, may be due to this difference. But even after taking this difference into consideration, the number of false positives predicted by our protocol turns out to be smaller than those predicted by the other two methods. For example, Figure 8 represents the case of argI and argF genes, where the NNPP program predicts a few extra TSS as compared to our method which correctly picks up a region in the vicinity of TSS. A combination of both the methods can therefore help in reducing the false predictions in the upstream and downstream regions. In principle, by restricting the pattern recognition using NNPP and Staden's methods only to the promoter region located initially with the help of our method, one can reduce the number of false positives. This composite approach will also help in pinpointing the TSS, which is not possible by use of our method alone. But at the same time it should be noted that both types of predictions fail to identify some of the promoters (Figure 8), e.g. for csiE gene, our program could correctly predict the promoter region but the NNPP program could not locate it. On the other hand, our program failed to find the promoter region for gyrA gene while NNPP could correctly position it. And in case of ilvA gene both the programs did not succeed in identifying the promoter region.

**Figure 9:**
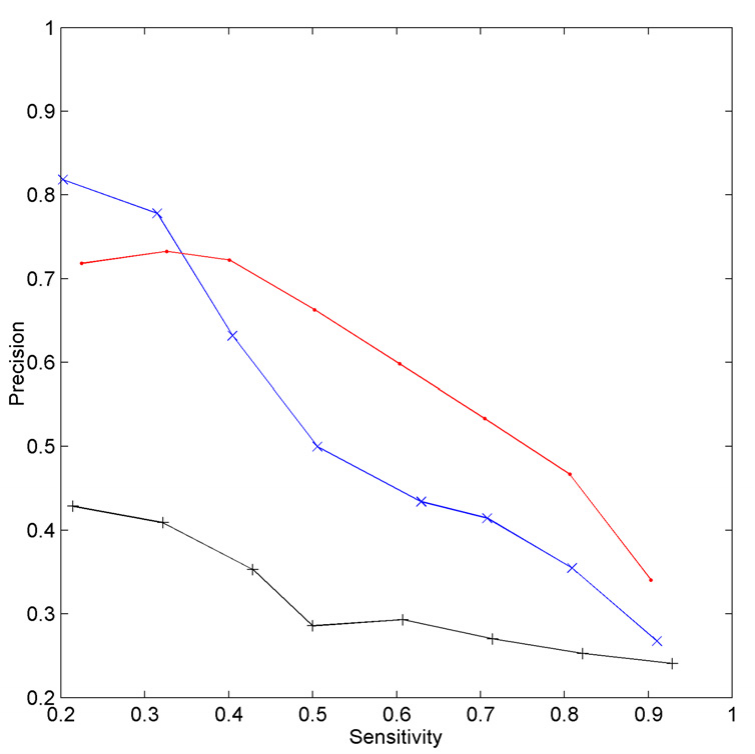
**Prediction accuracy of our method in case of promoters from different organisms **The precision (y-axis) of our method in predicting promoter region in different organisms *viz. Escherichia coli *(red), *Bacillus subtilis *(blue) and *Corynebacterium glutamicum *(black) is plotted against various levels of sensitivity (x-axis).

Very recently a study on improvement of NNPP prediction (TLS-NNPP), by combining this method with additional information such as distance between TSS and translation start site (TLS), has been published [25]. With the use of additional information regarding TLS, Burden *et al. *could significantly increase the precision of NNPP. The TLS-NNPP method was tested on 510 *E. coli *sequences of length 500 bp. For comparable sensitivity levels, the precision achieved by TLS-NNPP was 0.188 (sensitivity = 0.452) as compared to 0.109 precision (sensitivity = 0.443) achieved by NNPP. It can be seen that, for similar sensitivity levels, the precision achieved by our method (~0.7) is higher as compared to both TLS-NNPP and NNPP (Figure-9).

Presence of high densities of promoter like signals in the upstream region of TSS may be one of the reasons why pattern matching programs result in low level of precision. This has been shown recently by a systematic analysis of sigma70 promoters from *E. coli *[24]. In this study a number of weight matrices were generated by analysis of 599 experimentally verified promoters and these were tested on the 250 bp region upstream of gene start site. It was found that each 250 bp region on an average has 38 promoter-like signals. The study also presented a more rigorous patter searching method for locating promoters. With the use of this function the authors reach a sensitivity values of 0.86 but the corresponding precision achieved is only ~0.2. In case of our method, for a sensitivity of 0.9 we obtained a precision of 0.35 (as shown in Figure -9).

Recently Bockhorst *et al. *[26] proposed a very accurate method for predicting operons, promoters and terminators in *E. coli*. This method is based on sequence as well as expression data, but requires prior knowledge of coordinates of every ORF in the genome. We would like to emphasize here that our method is different from other methods in that it is independent of any such prior knowledge about the test gene or the organism and hence holds promise as being useful for promoter prediction in a newly sequenced genome.

The eukaryotic promoter prediction method proposed by Ohler *et al. *[27] is also worth mentioning here. Ohler *et al. *showed that a 30 % reduction of false positives can be achieved by use of physical properties, such as DNA bendability, in addition to other sequence properties of promoters. Interestingly, our method which also uses a physical property gives much smaller number of false positives as compared to Ohler *et al.*'s method. (For similar sensitivity, number of false predictions in case of Ohler *et al.*'s method are 1/4740 nt while in case of our method these are 1/8407 nt).

Another vertebrate promoter prediction program, 'Promfind' [28] identifies differences in hexanucleotide frequencies of promoter and coding region and is algorithmically quite similar to our method. But Promfind differs from our method in two important aspects. First, the Promfind program is developed mainly for vertebrate promoters and second, it assumes that in a given sequence, a promoter is always present and merely predicts its location. This need not necessarily be the case, as some of the sequences may not have any promoter at all. Our program differs from Promfind in that a promoter is predicted only when the sequence satisfies certain criteria and hence is much more appropriate for carrying out genome scale analysis.

### Promoter predictions in case of RNA genes

In addition to protein coding genes there are genes present for the non-coding RNAs (ncRNAs), which play structural, regulatory and catalytic roles. It is a difficult task to find out ncRNA genes in a genome because unlike protein coding regions they lack open reading frames and also they are generally smaller in size. In addition, it is also difficult to do a homology sequence search as only the structure of ncRNA is conserved and not the sequence. There are around 156 *E. coli *RNA genes reported on the NCBI site [29] and in addition many more small RNA genes are known to exist. Argaman *et al. *[30] recently identified 14 novel sRNA genes by applying a heuristic approach to search for transcriptional signals. We have checked the performance of our algorithm with respect to the 42 RNA transcription units (TUs) reported in Ecocyc database. Our method could pick up around 57 % RNA TUs, at a cut-off corresponding to 60 % sensitivity. The program works much better in case of rRNA operons than tRNA transcription units. We could correctly pick up promoter regions in 6 out of 7 rRNA transcription units, 17 out of 33 tRNA TUs and 1 out of the 2 remaining RNA types.

### Promoter prediction in *Bacillus subtilis *and *Corynebacterium glutamicum*

Finally, it is very important to see whether the method works equally well for other organisms which have genome compositions substantially different from that of *Escherichia coli*. Hence, we also tested our method using the promoter sequences from 1) the A+T-rich bacteria, *Bacillus subtilis *and 2) a G+C rich bacteria such as *Corynebacterium glutamicum*. Figure 9 gives a summary of the predictions in case of bacillus and corynebacterium promoters, along with those of *Escherichia coli*. It can be clearly seen that, at present our method performs optimally for the *Escherichia coli *promoters and also performs quite well in case of *Bacillus subtilis*. The prediction accuracy in case of *Corynebacterium glutamicum *promoters is not as good as that for the other two classes of promoters. However, it should be noted that the number of experimentally determined *Corynebacterium *promoters is much smaller as compared to other two bacteria and a larger dataset is required to arrive at any firm conclusion.

## Conclusions

It has often been suggested that use of certain properties of promoters, other than just the sequence motifs, which can distinguish promoters from other genomic regions, could significantly improve the gene prediction methods. Although the lower stability of promoter regions as compared to non-promoter regions has been reported previously, this observation was not incorporated into a promoter prediction program. We have been able to successfully use the differential stability of promoter sequences to predict promoter regions. Our method performs better as compared to currently available prokaryotic prediction methods and is also moderately successful in predicting RNA and bacillus promoter regions. The method certainly needs to be further improved to reduce the number of predicted false positives. This can be achieved by combining the approach presented here, with the earlier reported sequence analysis methods. Such a composite method will also help in pinpointing the TSS within the promoter region identified by our method.

## Methods

### Promoter sequence sets

All the promoter sequences used in this study are 1000 nt long, starting 500 nt upstream (position -500) and extending up to 500 nt downstream (position +500) of the TSS. In order to avoid having multiple TSS in a given 1000 nt sequence, we have excluded all the transcription start sites which are less than 500 nt apart. Our promoter set has 227 *E. coli *promoters, 89 *B. subtilis *promoters and 28 *C. glutamicum *promoters.

#### a) *Escherichia coli *promoter sequences

We tested our algorithm using the *Escherichia coli *promoter sequences, which were taken from the PromEC dataset [31]. The PromEC dataset provides a compilation of 471 experimentally identified transcriptional start sites. As mentioned above, after excluding all the transcription start sites which are less than 500 nt apart, the dataset contains 227 promoters. With the help of TSS information, promoter sequences were extracted from *Escherichia coli *genome sequence (NCBI accession no: NC_000913).

#### b) *Bacillus subtilis *promoter sequences

The transcription start sites for *Bacillus subtilis *promoters were obtained from the DBTBS database [32]. The required length sequences around transcription start sites were extracted from the Bacillus genome sequence (NCBI accession no: NC_000964).

#### c) *Corynebacterium glutamicum *promoter sequences

Analysis of *Corynebacterium glutamicum *promoters is carried out on a set of promoters compiled by Pàtek *et al. *[33] based on experimentally determined transcription sites.

#### d) RNA promoter sequences

The transcription start positions of RNA transcription units are obtained from the ecocyc dataset. In this set, both computer predicted as well as experimentally determined transcription start sites, are included. In total, we have 7 rRNA TUs, 33 tRNA TUs and 2 TUs of other RNAs.

### Free energy calculation

The stability of DNA molecule can be expressed in terms of free energy. The standard free energy change (ΔG^o^_37_) corresponding to the melting transition of an 'n' nucleotides (or 'n-1' dinucleotides) long DNA molecule, from double strand to single strand is calculated as follows:


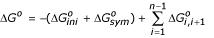


where,

ΔG^o^_ini _is the initiation free energy for dinucleotide of type ij.

ΔG^o^_sym _equals +0.43 kcal/mol and is applicable if the duplex is self-complementary.

ΔG^o^_i,j _is the standard free energy change for the dinucleotide of type ij.

Since our analysis involves long continuous stretches of DNA molecules, in our calculation we did not consider the two terms, ΔG^o^_ini _and ΔG^o^_sym_, which are more relevant for oligonucleotides. In the present calculation, each promoter sequence is divided into overlapping windows of 15 base pairs (or 14 dinucleotide steps). For each window, the free energy is calculated as given in the above equation and the energy value is assigned to the first base pair in the window. The energy values corresponding to the 10 unique dinucleotide sequences are taken from the unified parameters proposed recently [34,35].

### Statistical tests

#### a) Wilcoxon signed test for equality of medians

The free energy distribution at a given position, in the 1000 nt *E. coli *sequences ranging from -500 to +500, was compared to the distribution in a randomly selected set. For this comparison, we followed a similar procedure as adopted by Margalit *et al. *[11]. The random set was chosen such that an energy value per sequence was selected arbitrarily, independent of its position in the sequence. The comparison between the energy distributions was carried out using Wilcoxon signed test for equality of medians. This is a nonparametric test, which is used to test whether the two samples have equal medians or not.

#### b) Two-sample Kolmogorov-Smirnov test

We compared the free energy distribution at position -20 (with respect to TSS) with the distributions at the positions -200 and +200 using Kolmogorov-Smirnov two sample test [36].

All the calculations related to the statistical tests were carried out using MATLAB 6.0^®^.

### Implementation and scoring of NNPP and Staden's method

The promoter predictions were also carried out using two other methods *viz. *NNPP and Staden's method. NNPP program is available at [20]. All the NNPP predictions were carried out at a score cut-off 0.80.

The implementation of Staden's method was carried out as described in [21,37]. The weight matrix search was carried out with the help of PATSER program [38].

In case of NNPP as well as Staden's method, the true and false positives were scored as in case of our method (Figure 3), with a prediction in -150 to 50 region being considered as a true prediction.

### Sensitivity and precision

The sensitivity and precision for the predictions are calculated using the following formulae:


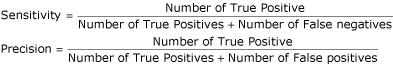


## Authors' contributions

AK performed the analysis, evaluated the results, and drafted the manuscript. MB suggested the problem, helped with evaluation of the results and the manuscript, also provided mentorship. All authors read and approved the final manuscript.

## Figures and Tables

**Table 1:** The number of false positives obtained for different levels of sensitivity.

Sensitivity	Cut-off for D	Cut-off for E1 (kcal/mole)	Frequency of false positives	
			
			FP (1/nt)^a^	FP (1/nt)^b^
0.13	3.4	-15.99	1/16214	1/261000
0.22	3.4	-16.7	1/11350	1/130500
0.32	3.3	-17.1	1/8407	1/65250
0.40	3.3	-17.55	1/6486	1/29000
0.50	2.76	-17.53	1/3914	1/13737
0.60	2.45	-17.64	1/2467	1/7250
0.70	2.35	-18.07	1/1621	1/2747
0.81	1.9	-18.15	1/1086	1/1878
0.90	0.97	-18.37	1/572	1/967

^a ^The false positives in the 1000 nt fragments, with TSS at the centre (-500 to +500).

^b ^The false positives in the 1000 nt fragments extracted from the centre of ORFs with length more than 2000 nt.

**Table 2:** Comparison of our method with other prokaryotic prediction algorithms vis-à-vis *Escherichia coli *promoters.

	TP	FP(1/nt)^a^	FP(1/nt)^b^
Our Program	195	1/780	1/1474
Neural Network [19]	195	1/233	1/514
Staden's method [21]	195	1/65	1/233

^a ^The false positives in the 1000 nt fragments with TSS at the centre (-500 to +500).

^b ^The false positives in the 1000 nt fragments extracted from the centre of ORFs with length more than 2000 nt.
